# *Tragia* L. Genus: Ethnopharmacological Use, Phytochemical Composition and Biological Activity

**DOI:** 10.3390/plants10122717

**Published:** 2021-12-10

**Authors:** Rodrigo Duarte-Casar, Juan Carlos Romero-Benavides

**Affiliations:** 1 Maestría en Química Aplicada, Facultad de Ciencias Exactas y Naturales, Universidad Técnica Particular de Loja, Loja 110108, Ecuador; rduarte@utpl.edu.ec; 2 Departamento de Química, Facultad de Ciencias Exactas y Naturales, Universidad Técnica Particular de Loja, Loja 110108, Ecuador

**Keywords:** *Tragia*, ethnopharmacology, phytochemicals, *Euphorbiaceae*, biological activities

## Abstract

*Tragia* L. is a genus of plants belonging to the *Euphorbiaceae* family with worldwide intertropical distribution, composed of more than 150 species. In this literature review, 26 species of the genus used as medicinal plants were found, mainly in East Africa and the Indian subcontinent, with a variety of uses among which antibacterial, anti-inflammatory, anticancer and reproductive health are most common. Research has been done on a few of the species, mostly those of the Old World, with emphasis on four of them: *Tragia involucrata* Linn., *Tragia spathulata* Benth., *Tragia benthamii* Baker and *Tragia plukenetii* Radcl.-Sm., confirming several ethnomedicinal claims. Moreover, a variety of active phytochemicals have been isolated, mainly ethers, hydrocarbons, flavonoids and sterols. There is ample field for the evaluation of the activity of *Tragia* extracts and essential oils and the identification of their active compounds, particularly of the New World species, for which there is still very little research.

## 1. Introduction

Plants have been used as a source of medicinal substances for a long time, with a use that amply predates history and presumably even mankind [1,2,3], and the discovery of active species and their use has historically been characterized by a trial-and-error approach [4]. This empirical knowledge has been and is being alidated by systematic research and is used as a guideline to direct the search for better and new drugs, integrating ancestral knowledge and modern methods [5].

Among the plant families considered medicinal, *Euphorbiaceae* is well regarded. The ample geographical distribution of the family and the variety of stress conditions the plants grow in, which trigger the production of secondary metabolites [6], partially explain the abundance and variety of biologically active compounds found in the family and thus its medicinal activity [7,8].

This review endeavors to summarize the current knowledge about species of the *Tragia* genus, which belongs to the *Euphorbiaceae* family, concerning their medicinal properties, phytochemical basis, and in vitro and in vivo evidence and envisioning future research prospects.

## 2. Genus

The genus *Tragia* is one of the 317 genera in the *Euphorbiaceae* family. There are 161 accepted names belonging to 154 species in the *Tragia* genus, with “pantropical and warm temperate distribution” [9,10]. The etymology for the name of this genus comes from the Greek *tragos*, meaning goat. This name may stem either from the name of the German botanist Hieronymus Bock—Bock means “ram” or “he-goat” in German, or from the hairy appearance of the plant that would resemble a male goat [11].

**Figure 1:**
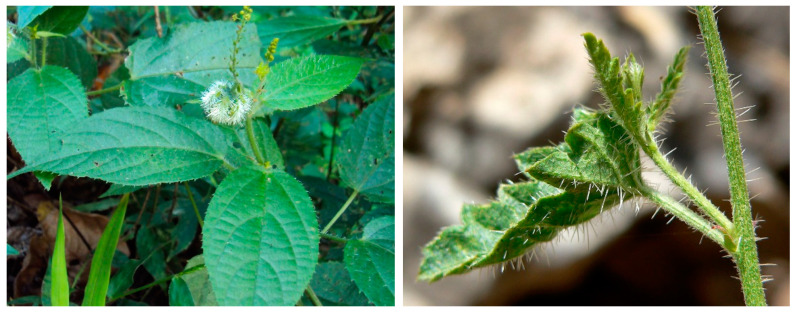
*Tragia involucrata* leaves, left. *Tragia ramosa* showing leaf and stem, covered by long, rough Scheme 3.0 license; right, Stan Shebs, GDFL license.

*Tragia* species exhibit very ample morphological characters: they are perennial plants with herb, shrub, subshrub and twining vine growth habits, with lanceolate leaves presenting either entire or serrated margins. Plants belonging to this genus sting when touched due to the presence of leaf hairs with a needle-shaped crystal of calcium oxalate (raphide) in the terminal cells that is expelled on contact and punctures the skin, allowing irritants to enter and cause transient stinging [12,13], presumably a defense mechanism against herbivores [14]. Several common names for Tragias, such as noseburn (*Tragia* spp.), Indian stinging nettle (*T. involucrata*), fireman (*T. volubilis*) or stinging nettle creeper (*T. durbanensis*), are due to this stinging property. Figure 1 shows *T. involucrata* leaf hairs with raphides visible, taken in Kerala, India, and *T. ramosa* with clearly visible raphides, taken in Nevada, USA.

Species belonging to *Euphorbiaceae* in general and to *Tragia* in particular are still not fully settled [8], as new species are being discovered [15] and species are being reassigned to other genera [9,16], so the number of species in the genus is still subject to change.

## 3. Distribution and Localization

**Figure 2:**
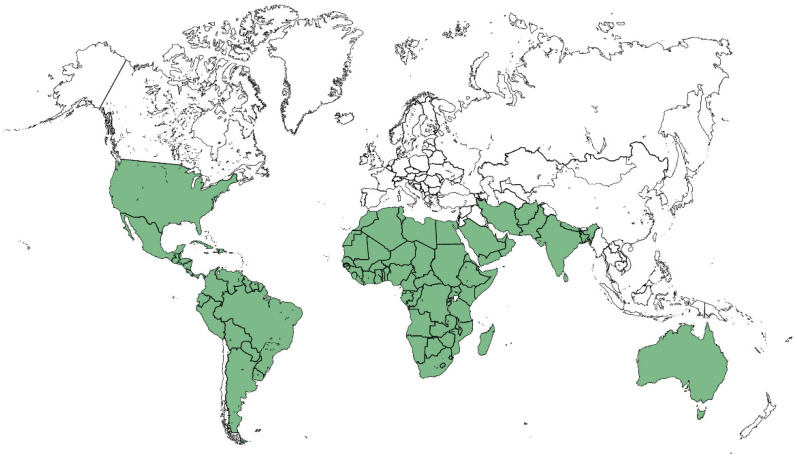
Worldwide Tragia species distribution, by country.

Species belonging to the *Tragia* genus are present in subtropical America, Eastern and Southern Africa, the Indian subcontinent and Northeastern Australia. Of the 154 species listed in the genus [17], 94 are found in Africa, 48 in America, 10 in Asia and 3 in Oceania, with some species such as *T. arabica* and *T. plukenetii* present both in Africa and Asia. The map in Figure 2 shows the intertropical distribution of *Tragia* species by country.

## 4. Methodology

Published works (articles and patents) were searched on scientific databases—Science Direct, Google Scholar and Scopus—for each species of the genus, using inverted commas for an exact match, e.g., “Tragia acalyphoides”. Relevant articles were selected after removing search terms unrelated to the area of interest such as corrosion, reforestation or hare diet. When abundant results were obtained, the search was refined with more specific terms, for example “Tragia involucrata medicinal” or “Tragia involucrata ethnopharmacology”. Duplicate articles were removed, and the remaining articles were reviewed with a focus on ethnopharmacological uses, phytochemical composition and biological activity, both in vitro and in vivo. When possible, the latest articles, no older than 10 years, have been cited. Preprints were not included.

**Figure 3:**
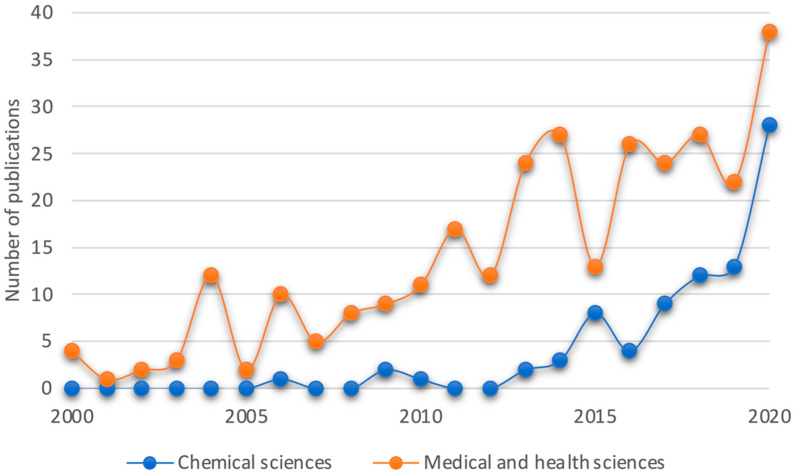
Publications containing the word *Tragia* since the year 2000 in Medical and Health sciences and in Chemical sciences. Data source: [18].

The research interest in *Tragia* species in medical and health sciences has increased during the last twenty years. Figure 3 shows the number of publications that include the word Tragia in their text in the fields mentioned. Even though the subject is not a very popular one, a steady increase in appearances can be seen, with a marked increase between 2019 and 2020 and the first half of 2021.

Compared to the other genera in the *Plukenieteae* tribe, *Tragia* concentrates 67% of the research, compared to 12% for *Cnesmone*, 10% for *Acidoton*, 4% for *Sphaerostylis* and 1% each for *Megistostigma*, *Pachystylidium*, *Platygyna* and *Tragiella* [18].

## 5. Ethnopharmacological Usage

Of the more than 150 species of the genus, few appear in the scientific literature, and even fewer are mentioned from an ethnopharmacological perspective. Notwithstanding, *Tragia* species are a part of traditional medicinal systems of East Africa and the Indian subcontinent, such as Siddha and Ayurveda [19], with documented uses of *T. involucrata* appearing as early as the 1st century CE [20] and with only a handful of mentions of *Tragia* species in the New World pharmacopoeia, concerning mostly topical applications. There is concern over an excessive use of *Tragia* species, e.g., *Tragia bicolor*, which poses a conservation hazard [21,22].

**Figure 4:**
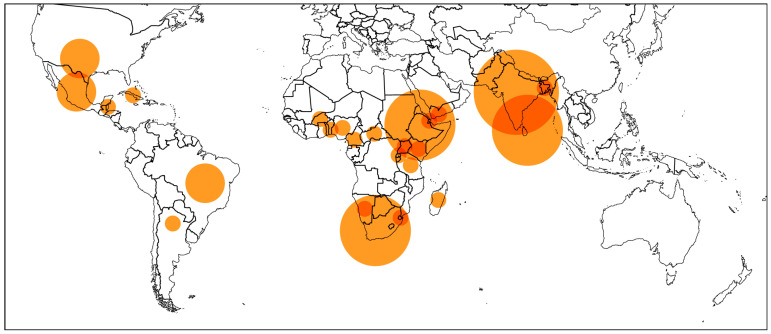
Ethnomedicinal uses for *Tragia* spp. The circle diameter is proportional to the uses reported for each country.

Most of the interest in this genus has been focused on four species: *Tragia involucrata*, *Tragia spathulata*, *Tragia plukenetii* and *Tragia benthamii* [23], with the bulk of the research focused on *T. involucrata*. Nevertheless, several more species and their medicinal uses appear in literature. Table 1 summarizes the species with reported medicinal use along with their stated ethnopharmacological uses, when available. The Anatomical Therapeutic Chemical (ATC) Classification by the World Health Organization (WHO) is used to classify the uses for each species [24]. Figure 4 shows the geographical distribution of the documented uses. The ethnomedical uses of *Tragia* spp are most abundant in the Indian subcontinent and East and Southern Africa.

According to the ATC classification, the most frequent ethnopharmacological uses of *Tragia* spp. in ethnopharmacology are: genitourinary system and sex hormones, with 19% of occurrences (15 of 77); nervous system, with 12%; and alimentary tract and metabolism, anti-infective for systemic use and antineoplastic and immunomodulating agents with 10% of occurrences each. The “various” classification presents 17% of occurrences, which include non-specified and vague uses, such as “toxic” or “medicinal”.

As for the morphological structures used per species, the most common are the leaves, 38%; followed by “not specified”, 33%; whole plant, 15%; roots, 13% and a single occurrence of endophytes (3%).

## 6. Biological Activity

Biological activity tests of *Tragia*, both in vitro and in vivo, are performed mostly with plant extracts and to a much lesser degree with essential oils: leaf, root or the whole plant, although ethnopharmacological uses mostly employ the plant via infusions, decoctions or ashes [23,35]. Different solvents and solvent mixtures have been used for the extracts, mainly methanol and ethanol. Due to the presence of *Tragia* in ethnomedical traditions in Africa and Asia, there is a team of research about the bioactivity of Old World *Tragia* extracts that have confirmed their activity and potency in some cases. Not all the health claims or traditional uses recorded have been validated through research. Again, the bulk of the research is centered on *T. involucrata*.

### 6.1. In Vitro Activity

**Figure 5:**
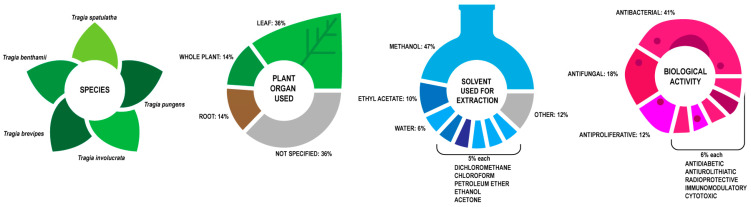
Summary of in vitro activity of Tragia species.

Extracts of *T. benthamii*, *T. brevipes*, *T. involucrata*, *T. pungens* and *T. spatulatha* have been tested to ascertain their in vitro activity for a variety of uses. The in vitro research is summarized in Figure 5.

Cases in which the efficacy has been shown in vitro are listed in Table 2.

In vitro biological activity tests devote the most attention to leaves (36%), with whole plant and root used to a lesser extent, with both 14%. Extraction solvents are methanol (47%), DCM (5%), Ethyl acetate (10%), water (6%), chloroform (5%), petroleum ether (5%), ethanol (5%) and acetone (5%). This solvent usage supports the assumption that most active compounds are moderately polar and are thus extracted with polar solvents.

Testing centers on antibacterial (41%) and antifungal (18%) activity of the extracts, with antiproliferative (12%) and antidiabetic, antiurolithiatic, radioprotective, immunomodulatory and cytotoxic effects (6% each) behind. This is a different profile than what was found in the ethnomedicinal claims, which centers on the genitourinary system and sex hormones. This is justified because aphrodisiacs do not have the expected properties [92].

### 6.2. In Vivo Activity

**Figure 6:**
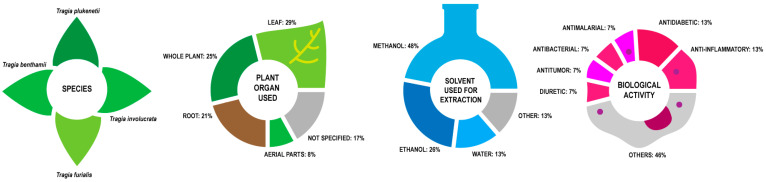
Summary of in vivo activity of *Tragia* extracts.

Besides in vitro activity testing, research has been done in animal models, mostly mice and also chicks, with at least one clinical trial performed in humans. The *Tragia* extracts evaluated in vivo, summarized in Figure 6 and Table 3, are obtained from four species: *T. benthamii*, *T. furialis*, *T. involucrata* and *T. plukenetii*.

Most of the research (73%) centers on *T. involucrata*, with *T. plukenetii* (18%), *T. benthamii* (9%) and *T. furialis* (5%) behind. In vivo assay extracts were obtained from leaves (29%), whole plant (25%), root (21%) and aerial parts (8%). Solvents used are methanol (48%), ethanol (26%) and water (13%), which shows that most active compounds are polar and are thus extracted with polar solvents.

For both in vitro and in vivo testing, the most common effect is antibacterial and antimicrobial with 22% of the reviewed studies. This is higher than the 10% reported in the ethnopharmacological uses. Effects having to do with cancer prevention and treatment—antiproliferative, antitumor, cytotoxic immunomodulatory and radioprotective—add up to 17% of the reported effects, which makes it the second most frequent use. Analgesic and anti-inflammatory activity is equally reported in 10% of the tests.

The findings reported in literature validate several medicinal use cases for *Tragia* species and dismiss some claims, e.g., *T. meyeriana* as an antineoplastic [60].

## 7. Phytochemical Composition

Phytochemical studies allow for the identification, separation and isolation of compounds of interest [109]. Based on phytochemical screenings published in the literature, the main secondary metabolites found in *Tragia* species extracts are alkaloids, glycosides, flavonoids, and sterols [23,110].

Some compounds found in plants belonging to the *Tragia* genus, classified according to their chemical nature, are listed in Table 4. Where applicable, the biological activity of the identified compound has been mentioned.

Identification of the compounds relies heavily on spectroscopic and spectrometric methods [109], and chromatography retention times and comparison with the literature are also used for tentative identification.

**Figure 7:**
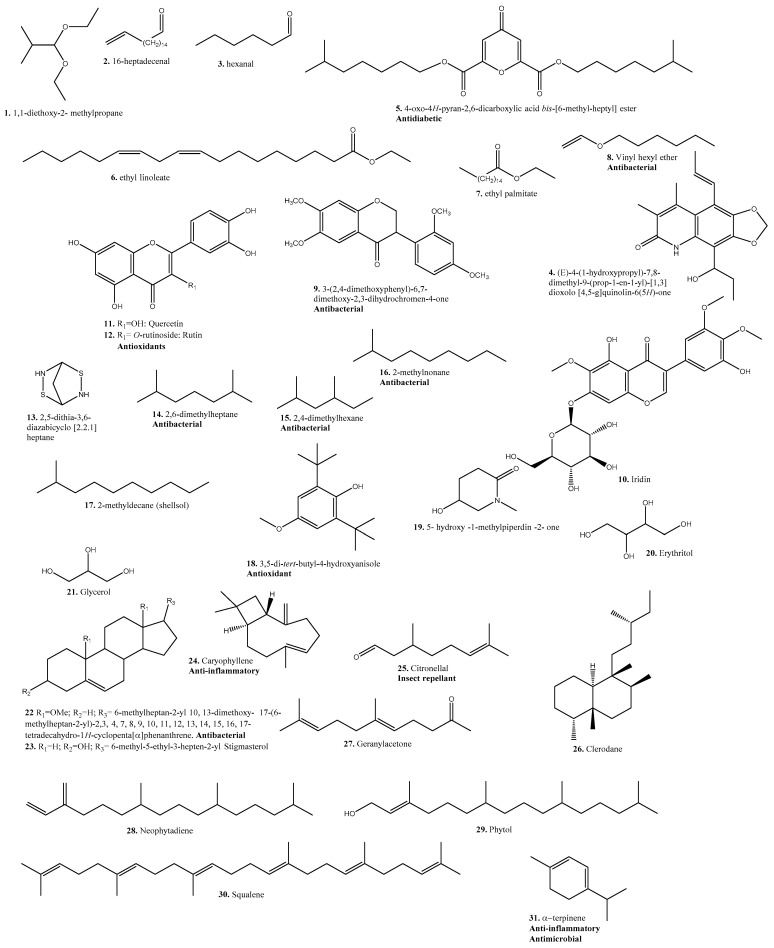
Compounds identified in *Tragia* extracts and oils.

Figure 7 shows the structure of some of the compounds identified in *Tragia* extracts and oils, mentioning their biological activity in bold when reported. As expected in plant extracts, there is a variety of secondary metabolites in the form of terpenoids and flavonoids. Ethers and non-terpenoid hydrocarbons are reported as having antibacterial activity, and they are not in any of the common groups of secondary metabolites. There is more information about the activity of the extracts and essential oils than about the activity of compounds on their own. The recent discovery of anti-inflammatory peptides in *Tragia benthamii* extracts [93] opens a new area of interest in the research of *Tragia* species.

A strength of the genus is its diversity and its pantropical distribution, which makes it readily available in most tropical countries. A weakness would be that, despite the interest shown concerning *T. involucrata* and other traditionally medicinal species, there appear to be no drugs derived from plants of these species, remaining in the realm of herbal remedies and plant extracts, entailing less medicinal interest than other genera of the *Euphorbiaceae* family, notably *Euphorbia* [8]. This can be attributed to the stage of research, with most work performed in vitro or in vivo and with a single clinical trial [52]. Hopefully the current research will advance into new drugs.

## 8. Conclusions

Species belonging to the *Tragia* genus are present in traditional medicine in several cultures and have multiple uses, among which antibacterial, anticancer and aphrodisiac are most frequent. There is scientific evidence that supports the use of these species in medicine, both at the extract level and at the active compound level, with in vivo tests in rats and mice, but there are no drugs derived from the species yet. The activity reported most frequently for *Tragia* extracts is antimicrobial and cancer-related, which suggests further research in those areas.

Less than 20% of the Tragia species are considered medicinal. This implies vast potential for screening and discovery of active compounds.

Most ethnopharmacological reports come from Asia and Africa, mainly East Africa and the Indian subcontinent. New world *Tragia* species have not been sufficiently studied and may prove to be a rich source of extracts and phytochemicals for drug research. Future directions for research include nanoparticles, the research into peptides extracted from *Tragia* species and the validation of medicines containing Tragia extracts against SARS-CoV-2.

## Figures and Tables

**Table 1:** *Tragia* species and their ethnopharmacological use. Species are listed in alphabetical order and validated against [25].

Species	Region	Plant Organs Used	Use	Form of Usage	ATC Category	References
*Tragia aliena Pax and K.Hoffm.*	Brazil	NS	Medicinal (not specified)	NS	V	[26]
*Tragia benthamii* Baker	Nigeria, Cameroon	Whole plant Leaves, roots Whole plant	Abortifacient Antimalarial Filaricidal	Decoction NS	G P P	[27] [28] [23]
*Tragia bicolor* Miq.	India, Sri Lanka	NS	Medicinal	NS	V	[21]
*Tragia brevipes* Pax.	Rwanda, Kenya	Leaves	Anticancer Antigonorrhoeic Aphrodisiac Erectile dysfunction Obesity Uterotonic	Decoction Chewing Ash	L G G G A G	[29] [30] [31] [32] [33] [34] [35] [36]
*Tragia cinerea (Pax) M.G.Gilbert and Radcl.-Sm.*	Ethiopia	Leaves NS	Antigonorrhoeic Anti-inflammatory Aphrodisiac	Powdered plant, drunk mixed with butter/honey	G M G	[37] [38]
*Tragia cordata* Michx.	America, Ethiopia	Roots	Urinary tract and external parasites	Decoction Topical (powdered root)	G D	[39]
*Tragia dioica* Sond.	South Africa	Leaves	Fatigue Tuberculosis	NS	V J	[40]
*Tragia doryodes M.G. Gilbert*	Ethiopia	Leaves	Anthrax	Decoction	J	[41]
*Tragia durbanensis* Kuntze.	South Africa	NS	Skin rashes	NS	D	[42]
*Tragia furialis Bojer*	Tanzania, Madagascar	Roots	Abscess Analgesic Antimalarial Aphrodisiac Paralysis	Cold water maceration, drunk	J N P G N	[43] [44] [45]
*Tragia geraniifolia Klotzsch ex Müll.Arg.*	Argentina	Roots NS	Emollient Rubefacient Diuretic Antirheumatic	NS	D D G M	[46] [47]
*Tragia gracilis* Griseb.	Cuba	NS	Not specified	NS	V	[48]
*Tragia hildebrandtii Müll.Arg.*	India	NS	Not specified	NS	V	[49]
*Tragia hispida* Willd.	Sri Lanka	NS	Tooth decay	NS	A	[50]
*Tragia insuavis* Prain.	Kenya	Endophytes	Antibacterial	NS	J	[51]
*Tragia involucrata* L.	Southern Asia (India, Sri Lanka, Bangladesh)	Whole plant, Leaves, Roots	Analgesic Antidiabetic Anti-inflammatory Antimicrobial Antinociceptive Antioxidant Antiparasitic Antitumor Diuretic Hepatoprotective	Decoction Juice Poultice	N A M J N – D L G N	[20,52] [53] [23] [54] [55] [56] [57] [58]
*Tragia meyeriana Müll.Arg.*	South Africa	NS Leaves, Stems NS (barks, stems and corms mentioned)	Aphrodisiac Antineoplastic Immune booster	Decoction	G L L	[59] [60] [61]
*Tragia mitis Hochst. ex A.Rich.*	Ethiopia	Root	Antidiarrheal	Crushed, mixed with water and sugar	A	[62]
*Tragia mixta* M.G.Gilbert	Djibouti	Leaves	Analgesic Stomach aches Tonsilitis	Heated Poultice	N A A	[63] [64]
*Tragia okanyua* Pax	Namibia	NS Root	Dizziness Snake bite Cardiovascular problems Sexually transmitted diseases (STD)	Powdered, drunk with water	N V B G	[65] [66]
*Tragia plukenetii Radcl.-Sm.*	East Africa, India	Leaves	Antihyperglycemic Antitumor	Decoction	A L	[23]
*Tragia praetervisa* Chakrab. & N.P.Balakr.	India, Sri Lanka	NS	Not specified	NS	V	[49]
*Tragia preussii* Pax	Central African Republic	Leaves	Rheumatism	NS	M	[67]
*Tragia pungens* (Forssk.) Müll.Arg.	Yemen	Whole plant	Allergy and skin diseases Antirheumatic Cytotoxic Anti-impotence	Paste	D M L G	[68] [69] [70]
*Tragia ramosa* Torr.	U.S.A., Mexico	Leaves	Not specified	NS	V	[71]
*Tragia rupestris* Sond.	South Africa	Whole plant	Medicine (not specified)	NS	V V	[72] [73]
*Tragia senegalensis* Müll. Arg	Benin	Leaves	Azoospermia	NS	G	[74]
*Tragia sonderi* Prain	Swaziland	Root	HIV/AIDS	Decoction Topical	L	[75]
*Tragia spathulata* Benth.	West Africa	Leaves	Antibacterial	NS	J	[23] [76]
*Tragia subsessilis* Pax	Uganda	Root	Tuberculosis	NS	J	[77]
*Tragia uberabana* Müll. Arg	Brazil	NS	Medicinal Toxic	NS	V V	[78]
*Tragia vogelii* Keay	Burkina Faso	Whole plant	Abortifacient	Decoction	G	[79]
*Tragia volubilis* L.	Mexico, Antilles, Brazil	Leaves, Stem, Root	Diuretic Medicinal STDs	Decoction	G V G	[80] [26] [46,81]
*Tragia yucatanensis* Millsp.	Belize, Guatemala, Mexico	Leaves	Burns Rheumatism	Topical	D M	[82]

NS: not specified. ATC categories are as follows. A: alimentary tract and metabolism, B: blood and blood-forming organs, C: cardiovascular system, D: dermatological, G: genitourinary system and sex hormones, H: systemic hormonal preparations, excluding sex hormones and insulins, J: anti-infective for systemic use, L: antineoplastic and immunomodulating agents, M: musculo-skeletal system, N: nervous system, P: antiparasitic products, insecticides and repellents; R: respiratory system, S: sensory organs; V: various [24], not present in the classification. STDs: sexually transmitted diseases.

**Table 2:** In vitro activity of *Tragia* extracts. Species are in alphabetical order.

Species	Extract	Plant Organs Used	Biological Activity	Biological Model	Effect	Methodology	Reference
*T. benthamii*	Methanol	Whole plant	Antibacterial	28 strains (sensitive and MDR) of *Pseudomonas aeruginosa,* *Klebsiella pneumoniae,* *Enterobacter aerogenes,* *Escherichia coli,* *Providencia stuartii*	Effective against 11/28 strains (39.3%)	256–1024 μg/mL INT colorimetric assay	[83]
*T. brevipes*	Methanol: water 9:1	Leaf	Antibacterial		Inhibition zones (mm)	500 mg/mL extract—well diffusion assay	[84]
				*Escherichia coli,*	+2		
				*Salmonella* spp.,	+10		
				*Enterobacter aerogenes,*	+9		
				*Bacillus cereus,*	+24		
				*Serratia liquefaciens,*	+5		
				*Proteus vulgaris*	+8		
*T. brevipes*	Methanol:DCM 1:1	Leaf	Antiproliferative	DU145	+IC_50_: 30 μg/mL	Extract MTT	[85]
				HCC	-		
				HELA	-		
*T. involucrata*	Chloroform	Root	Antidiabetic	Fertile eggs of white leghorn chicken	+	0.5, 1 mg/egg. Streptozotocin-induced diabetes	[86]
*T. involucrata*	Ethyl acetate	Root	Antibacterial Antifungal		Inhibition zones (mm)	50–250 mg/mL. Disc diffusion	[53]
				*Staphylococcus aureus*	+18		
				*Bacillus subtilis*	+14		
				*Bacillus brevis*	+5.7		
				*Staphylococcus epidermidis*	+0.6		
				*Escherichia coli*	+17		
				*Shigella disenteriae*	+3.7		
				*Pseudomonas aeruginosa*	+9.4		
				*Vibrio cholera*	+4.7		
				*Trichophyton rubrum*	+3.7		
				*Malassezia furfur*	+13.5		
*T. involucrata*	Methanol	Leaf	Antifungal		Inhibition zone	Agar disc diffusion	[87]
				*Rhizopus stolonifer,*	+16 ± 0.3 mm		
				*Aspergillus niger,*	+15 ± 0.2 mm		
				*Alternaria solani,*	+15 ± 0.6 mm		
				*Mucor indicus,*	-		
				*Chaetomium globosum,*	-		
				*Tilletia indica*	+10 ± 0.5 mm		
*T. involucrata*	Isolated hydrocarbons and ethers	-	Antibacterial		Inhibition zone mm	Agar disc diffusion	[88]
				*Burkholderia pseudomallei (TES21),*	+23		
				*Burkholderia pseudomallei (KHW),*	+25		
				*Klebsiella pneumoniae (ATCC15380)*	-		
				*Klebsiella pneumoniae*	+20		
				*Pseudomonas aeruginosa (ATCC27853),*	-		
				*Vibrio damsela,*	+19		
				*Salmonella typhi (ATCC51812)*	+28		
*T. involucrata*	Methanol Ethyl acetate Chloroform Petroleum ether	Leaf	Antiproliferative	K562 cell lines	- CHCl_3_ - AcOEt	MTT	[89]
*T. involucrata*	Water +NP	Leaf	Antiurolithiatic	-	+Struvite crystal growth inhibitory effect	2% extract; AgNPs (200 μg mL^−1^)	[90]
*T. involucrata*	Methanol	Whole plant	Radioprotective	Cultured human peripheral lymphocytes	+Pretreatment (10 μg mL^−1^)	^60^Co gamma irradiation Comet assay	[91]
*T. meyeriana* and other plant species	Boiling water	Whole plant	Immunomodulatory	Isolated peripheral blood mononuclear cells	+	*S. aureus* stimulation. Inflammatory cytokine secretion in THP-1 monocytes	[61]
*T. pungens*	Methanol	NS	Antibacterial Cytotoxic	*Staphylococcus aureus*	+(8–14 mm)	Disk diffusion assay, Neutral red uptake assay	[69]
				*Bacillus subtilis*	-		
				*Micrococcus flavus*	-		
				*Pseudomonas aeruginosa*	-		
				*Candida maltosa*	-		
				*FL cells*	+Cytotoxicity. IC_50_: 70 μg/mL		
*T. spatulatha*	Ethanol Methanol Acetone	Leaf	Antibacterial Antifungal		MIC (mg/mL)	Agar well diffusion	[76]
				*Staphylococcus aureus,*	+21		
				*Proteus mirabilis,*	+21		
				*Klebsiella pneumoniae,*	+25		
				*Salmonella typhi,*	+25		
				*Streptococcus pneumoniae,*	+25		
				*Escherichia coli,*	-		
				*Candida albicans,*	-		
				*Aspergillus flavus,*	-		
				*Fusarium solani*	-		

MDR: multi-drug resistant. NP: nanoparticle. DCM: dichloromethane. NS: not specified; INT: p-Iodonitrotetrazolium chloride; MTT: 3-(4-5-dimethyl-2-thiazoly)-2,5-diphenyltetrazolium bromide; MIC; minimum inhibitory concentration; AcOEt: ethyl acetate; AgNP: silver nanoparticles; + active. - not active.

**Table 3:** In vivo activity of *Tragia* extracts.

Species	Extract	Plant Organs Used	Animal Model	Activity	Results	Reference
*T. benthamii*	Ethanol	Whole plant	Swiss albino mice	Antimalarial	−Very poor activity against *P. berghei* (NK-65) at 50 mg·kg^−1^ bw.	[27]
*T. benthamii*	Water	NS	Chick	Anti-inflammatory	+Carrageenan-induced foot edema. Maximal inhibition 84.3% at 300 mg/kg bw.	[93]
*T. furialis*	Ethanol–water	NS	White albino mice	Antimalarial	+IC_50_: 639.3 mg·kg^−1^ bw against *P. berghei*.	[43]
*T. involucrata*		Root	Wistar rats	Hepatoprotective	+100–300 mg/kg bw. Hepatoprotective against CCl_4_ induced toxicity and antioxidant activity; Attenuation of biomarker alteration (SGOT, SGPT, ALP. TP).	[57]
*T. involucrata*	Benzene: Ethyl acetate 1:1	Root	*Culex quinquefasciatus*	Larvicidal	+0.1–0.4% *w*/*v* Oviposition and phagodeterrence, larvicidal.	[94]
*T. involucrata*	Ethanol	Leaf	Albino rats (male)	Nephroprotective	+250 and 500 mg/kg bw. Decrease in serum urea and creatinine in acetaminophen-induced toxicity.	[95]
*T. involucrata*	Hexane Ethyl acetate	Aerial parts	Swiss albino mice	Antitumor	+50–150 mg/kg bw. Ehrlich’s Ascites Carcinoma. DD antitumor activity and increased life span for both extracts.	[96]
*T. involucrata*	Hot water	NS	Wistar rats (male)	Diuretic	+1650, 2200 mg/kg bw. Loop diuretic action.	[56]
*T. involucrata*	Hot water—freeze dried	Whole plant	Clinical trial	Antidiabetic	240 mL decoction/day. FPG decrease from 164.4 ± 20.4 to 130.9 ± 16.2 mg/dL.	[52]
*T. involucrata*	Methanol	Leaf	Swiss albino mice	Analgesic Anxiolytic Sedative	+200, 400 mg/kg bw. Acetic acid writhing and formalin-induced paw licking; behavioral tests; pentobarbital-induced sleep time.	[97]
*T. involucrata*	Methanol	Leaf	Wistar rats	Antibacterial	+100, 200 mg/kg bw. Wound healing in *S. aureus* infections.	[98]
*T. involucrata*	Methanol	Leaf	Swiss albino mice	Antiepileptic	+400, 800 mg/kg bw MES, PTZ, PTX induced convulsions DD.	[99]
*T. involucrata*	Methanol	NS	Swiss albino mice	Radioprotective	+100 mg/kg bw. DD survival increase	[100]
*T. involucrata*	Methanol	Root	Charles-Foster rats Swiss albino mice	Analgesic Anti-inflammatory	+Carrageenan paw edema, cotton pellet granulomata, acetic acid writhing.	[101]
*T. involucrata*	Methanol	Root	Wistar rats	Antibacterial	+100, 200 mg/kg bw. Wound healing in *S. aureus* infections	[102]
*T. involucrata*	Methanol	Root	Charles−Foster rats Swiss albino mice	CNS depressant	+100–300 mg/kg bw. Behavioral pattern, spontaneous motility, pentobarbitone-induced sleep, body temperature, aggressive behavior pattern and conditioned avoidance response (CAR).	[103]
*T. involucrata*	Methanol Chloroform	Whole plant	Albino rats	Anti-inflammatory	+100, 300 mg/kg bw. Both extracts. Carrageenan paw oedema.	[54]
*T. involucrata*	Methanol Ethyl acetate	Whole plant	Swiss albino mice	Analgesic	+500 mg/kg bw. Acetic acid model; tail flick model analgesic activity.	[55]
*T. involucrata*	Water	Leaf	Wistar rats Swiss mice (male)	Anti-inflammatory	+50–400 mg/kg bw in carrageenan-induced hindpaw edema and cotton pellet granuloma models.	[104]
*T. involucrata*	Water +NP	Leaf	Wistar rats (male)	Antiurolithiatic	+200 mg/kg bw. CaOx stone formation inhibition in ethylene glycol-induced urolithiasis.	[90]
*T. plukenetii*	Ethanol	Aerial parts	Wistar rats (male)	Antihyperglycemic	+At an oral dose of 150 and 300 mg/kg bw. Oral glucose tolerance test in alloxan induced diabetic rats.	[105]
*T. plukenetii*	Ethanol	Whole plant	Wistar rats Guinea pigs Rabbits	Antipyretic Diuretic Antiasthmatic Analgesic Antispasmodic	+100 mg/kg bw. +Antipyretic: Brewer’s yeast-induced hyperpyrexia method. +Diuretic: in vivo Lipschitz test method. +Antiasthmatic: Isolation of guinea pig ileum preparation; histamine-induced bronchoconstruction. +Analgesic: acetic acid writhing response. +Antispasmodic: studies on isolated rabbit jejunum.	[106]
*T. plukenetii*	Ethanol	Whole plant	Swiss albino mice (male)	Antitumor	+100–300mg/kg bw. Ehrlich ascites carcinoma survivability. Antioxidant parameters increased DD.	[107]
*T. plukenetii*	Methanol Benzene Chloroform	Leaf	Swiss albino mice	Anticonvulsant	+100 mg/kg bw. Methanol extract against PTZ-induced convulsions.	[108]

NS: not specified; −: no activity; +: activity present; DD: dose-dependent, bw: body weight; MES: maximal electroshock; PTZ: pentylenetetrazol; PTX: picrotoxin; FPG: fasting plasma glucose; SGOT: serum glutamic oxaloacetic transaminase; SGPT: serum glutamic pyruvic transaminase; ALP: alkaline phosphatase.

**Table 4:** Compounds isolated/identified in *Tragia* extracts and oils and their biological effect.

No.	Compound	Identified	Isolated	Methodology Used	Species	Collection area	Plant Organ Used	Use	Effect	Reference
**Acetal**										
1	1,1-diethoxy-2- methylpropane	X		Ethanol extract GC, MS	*T. plukenetii*	NS	Whole plant	NS	NS	[111]
				Aldehydes						
2	16-heptadecenal	X		Ethanol extract GC, MS	*T. plukenetii*	NS	Whole plant	NS	NS	[111]
3	Hexanal	X		Hydrodistillation GC/GC-MS	*T. benthamii*	Ibadan, Nigeria	Leaves	NS	NS	[112]
				Alkaloid						
4	(E)-4-(1-hydroxypropyl)-7,8-dimethyl-9-(prop-1-en-1-yl)-[1,3] dioxolo [4,5-g]quinolin-6(5*H*)-one	X	X	Acidified ethanol extract GC, MS, LC	*T. plukenetii*	NS	Whole plant	NS	NS	[111]
**Esters**										
5	4-oxo-4*H*-pyran-2,6-dicarboxylic acid *bis*-[6-methyl-heptyl] ester	X	X	Ethanol extract IR ^1^H, ^13^C NMR, MS	*T. involucrata*	Salem, India	Roots	Antidiabetic	Blood glucose reduction	[86]
6	Ethyl linoleate	X	X	Ethanol extract GC, MS	*T. plukenetii*	NS	Whole plant	NS	NS	[111]
7	Ethyl palmitate	X	X	Ethanol extract GC, MS	*T. plukenetii*	NS	Whole plant	NS	NS	[111]
				Ether						
8	Vinyl hexyl ether	X	X	Aqueous extract GC, MS	*T. involucrata*	Tamil Nadu, India	Leaf	Antibacterial *Escherichia coli* *Proteus vulgaris* *Staphylococcus aureus*	MBC 12.25 μg/mL	[98,113]
**Flavonoids**										
9	3-(2,4-dimethoxyphenyl)-6,7-dimethoxy-2,3-dihydrochromen-4-one	X	X	Ethyl acetate extract FTIR, MS, ^1^H NMR	*T. involucrata*	Odisha, India	Root	Antibacterial Fungicidal	MIC 1.25-12.5 μg/mL	[53]
10	Iridin	X	X	Ethyl acetate extract FTIR, MS, ^1^H NMR	*T. involucrata*	Odisha, India	Root	Toxic		[53]
11	Quercetin	X	X	Ethyl acetate extract FTIR, MS, ^1^H NMR	*T. involucrata*	Odisha, India	Root	Antioxidant		[53]
12	Rutin	X	X	Ethyl acetate extract FTIR, MS, ^1^H NMR	*T. involucrata*	Odisha, India	Root	Antioxidant		[53]
**Heterocycle**										
13	2,5-dithia-3,6-diazabicyclo[2.2.1]heptane	X	X	95% aqueous ethanol extraction ^1^H, ^13^C NMR	*T. benthamii*	Ibadan, Nigeria	Whole plant	NS		[114]
**Hydrocarbons**										
14	2,6-dimethylheptane	X	X	Aqueous extract GC, MS	*T. involucrata*	Tamil Nadu, India	Leaf	Antibacterial *Proteus vulgaris*	MBC 10 μg/mL	[98]
15	2,4-dimethylhexane	X	X	Aqueous extract GC, MS	*T. involucrata*	Tamil Nadu, India	Leaf	Antibacterial *Staphylococcus aureus*	MBC 12.25 μg/mL	[98]
16	2-methylnonane	X	X	Aqueous extract GC, MS	*T. involucrata*	Tamil Nadu, India	Leaf	Antibacterial *Escherichia coli* *Proteus vulgaris* *Staphylococcus aureus*	MIC 5.0 μg/mL	[98]
17	Shellsol (2-methyldecane)	X	X	Aqueous extract GC, MS	*T. involucrata*	Tamil Nadu, India	Leaf	Antibacterial *Proteus vulgaris* *Staphylococcus aureus*	MBC 25.0 μg/mL	[98]
18	3,5-di-*tert*-butyl-4-hydroxyanisole	X	X	95% aqueous ethanol extraction ^1^H, ^13^C NMR	*T. benthamii*	Ibadan, Nigeria	Whole plant	Antioxidant		[114]
19	5-hydroxy-1-methylpiperdin-2-one	X	X	Methanol extract IR, ^1^H, ^13^C RMN, LC	*T. involucrata*	Kerala, India	Leaf	Antihistamine	Muscle relaxant, bronchodilating and anti-allergic effects	[115]
**Polyols**										
20	Erythritol	X	X	95% aqueous ethanol extraction ^1^H, ^13^C NMR	*T. benthamii*	Ibadan, Nigeria	Whole plant	NS	NS	[114]
21	Glycerol	X	X	95% aqueous ethanol extraction ^1^H, ^13^C NMR	*T. benthamii*	Ibadan, Nigeria	Whole plant	NS	NS	[114]
**Terpenoids**										
22	10,13-dimethoxy-17-(6-methylheptan-2-yl)-2,3,4,7,8,9,10,11,12,13,14,15,16,17-tetradecahydro-1*H*-cyclopenta[α]phenanthrene.	X	X	Ethyl acetate extract FTIR, MS, ^1^H NMR	*T. involucrata*	Odisha, India	Root	NS	NS	[53]
23	Stigmasterol	X		Aqueous extract GC, MS	*T. involucrata*		Leaf	NS	NS	[98]
24	Caryophyllene	X		Hydrodistillation GC/GC-MS	*T. benthamii*	Ibadan, Nigeria	Leaves	Anti inflammatory		[112]
25	Citronellal	X	X	Ethanol extract IR, ^1^H RMN, LC	*T. ramosa*	Maharashtra, India	Leaves	Antibacterial		[71]
26	Clerodane	X	X	Ethanol extract IR, ^1^H RMN, LC	*T. ramosa*	Maharashtra, India	Leaves	NS	NS	[71]
27	Geranylacetone	X		Hydrodistillation GC/GC-MS	*T. benthamii*	Ibadan, Nigeria	Leaves	NS	NS	[112]
28	Neophytadiene (2-(4,8,12-Trimethyltridecyl) buta-1,3-diene)	X	X	Ethanol extract GC, MS	*T. plukenetii*	NS	Whole plant	NS	NS	[111]
29	Phytol	X	X	95% aqueous ethanol extraction ^1^H, ^13^C NMR	*T. benthamii*	Ibadan, Nigeria	Whole plant	NS	NS	[114]
30	Squalene (all *trans*)	X	X	Ethanol extract GC, MS	*T. plukenetii*	NS	Whole plant	NS	NS	[111]
31	α-terpinene	X	X	Ethanol extract IR, ^1^H RMN, LC	*T. ramosa*	Maharashtra, India	Leaves	Antiinflammatory, Antimicrobial	NS	[71]

GC: gas chromatography; MS: mass spectrometry; LC: liquid chromatography; IR: infrared spectroscopy; NMR: nuclear magnetic resonance; FTIR: Fourier transform infrared spectroscopy; Q-TOF: quadrupole time of flight mass spectrometry; TLC: thin layer chromatography; NS: not specified.
